# Reply to: Multivariate BWAS can be replicable with moderate sample sizes

**DOI:** 10.1038/s41586-023-05746-w

**Published:** 2023-03-08

**Authors:** Brenden Tervo-Clemmens, Scott Marek, Roselyne J. Chauvin, Andrew N. Van, Benjamin P. Kay, Timothy O. Laumann, Wesley K. Thompson, Thomas E. Nichols, B. T. Thomas Yeo, Deanna M. Barch, Beatriz Luna, Damien A. Fair, Nico U. F. Dosenbach

**Affiliations:** 1 Department of Psychiatry, Massachusetts General Hospital, Harvard Medical School, Boston, MA USA; 2 Department of Radiology, Washington University School of Medicine, St Louis, MO USA; 3 Department of Psychiatry, Washington University School of Medicine, St Louis, MO USA; 4 Department of Neurology, Washington University School of Medicine, St Louis, MO USA; 5 Department of Biomedical Engineering, Washington University in St Louis, St Louis, MO USA; 6 Division of Biostatistics, University of California San Diego, La Jolla, CA USA; 7 Oxford Big Data Institute, Li Ka Shing Centre for Health Information and Discovery, Nuffield Department of Population Health, University of Oxford, Oxford, UK; 8 Department of Electrical and Computer Engineering, National University of Singapore, Singapore, Singapore; 9 Centre for Sleep and Cognition, National University of Singapore, Singapore, Singapore; 10 Centre for Translational MR Research, National University of Singapore, Singapore, Singapore; 11 N.1 Institute for Health, Institute for Digital Medicine, National University of Singapore, Singapore, Singapore; 12 Integrative Sciences and Engineering Programme, National University of Singapore, Singapore, Singapore; 13 Martinos Center for Biomedical Imaging, Massachusetts General Hospital, Charlestown, MA USA; 14 Department of Psychological and Brain Sciences, Washington University in St Louis, St Louis, MO USA; 15 Department of Psychology, University of Pittsburgh, Pittsburgh, PA USA; 16 Department of Psychiatry, University of Pittsburgh, Pittsburgh, PA USA; 17 Masonic Institute for the Developing Brain, University of Minnesota Medical School, Minneapolis, MN USA; 18 Department of Pediatrics, University of Minnesota Medical School, Minneapolis, MN USA; 19 Institute of Child Development, University of Minnesota Medical School, Minneapolis, MN USA; 20 Program in Occupational Therapy, Washington University School of Medicine, St Louis, MO USA; 21 Department of Pediatrics, Washington University School of Medicine, St Louis, MO USA

**Keywords:** Cognitive neuroscience, Neuroscience

replying to: T. Spisak et al. *Nature* 10.1038/s41586-023-05745-x (2023)

In our previous study^1^, we documented the effect of sample size on the reproducibility of brain-wide association studies (BWAS) that aim to cross-sectionally relate individual differences in human brain structure (cortical thickness) or function (resting-state functional connectivity (RSFC)) to cognitive or mental health phenotypes. Applying univariate and multivariate methods (for example, support vector regression (SVR)) to three large-scale neuroimaging datasets (total *n* ≈ 50,000), we found that overall BWAS reproducibility was low for *n* < 1,000, due to smaller than expected effect sizes. When samples and true effects are small, sampling variability, and/or overfitting can generate ‘statistically significant’ associations that are likely to be reported due to publication bias, but are not reproducible^2–5^, and we therefore suggested that BWAS should build on recent precedents^6,7^ and continue to aim for samples in the thousands. In the accompanying Comment, Spisak et al.^8^ agree that larger BWAS are better^5,9^, but argue that “multivariate BWAS effects in high-quality datasets can be replicable with substantially smaller sample sizes in some cases” (*n* = 75–500); this suggestion is made on the basis of analyses of a selected subset of multivariate cognition/RSFC associations with larger effect sizes, using their preferred method (ridge regression with partial correlations) in a demographically more homogeneous, single-site/scanner sample (Human Connectome Project (HCP), *n* = 1,200, aged 22–35 years).

There is no disagreement that a minority of BWAS effects can replicate in smaller samples, as shown with our original methods^1^). Using the exact methodology (including cross-validation) and code of Spisak et al.^8^ to repeat 64 multivariate BWAS in the 21-site, larger and more diverse Adolescent Brain Cognitive Development Study (ABCD, *n* = 11,874, aged 9–11 years), we found that 31% replicated at *n* = 1,000, dropping to 14% at *n* = 500 and none at *n* = 75. Contrary to the claims of Spisak et al.^8^, replication failure was the outcome in most cases when applied to this larger, more diverse dataset. Basing general BWAS sample size recommendations on the largest effects has at least two fundamental flaws: (1) failing to detect other true effects (for example, reducing the sample size from *n* = 1,000 to *n* = 500 leads to a 55% false-negative rate), therefore restricting BWAS scope, and (2) inflation of reported effects^3,10–12^. Thus, regardless of the method, associations based on small samples can remain distorted and lack generalizability until confirmed in large, diverse, independent samples.

We always test for BWAS replication with null models (using permutation tests) of out-of-sample estimates to ensure that our reported reproducibility is unaffected by in-sample overfitting. Nonetheless, Spisak et al.^8^ argue against plotting inflated in-sample estimates^1,10^ on the *y* axis, and out-of-sample values on the *x* axis, as we did (Fig. 1a). Instead, they propose plotting cross-validated associations from an initial, discovery sample (Fig. 1b (*y* axis)) against split-half out-of-sample associations (*x* axis). However, cross-validation—just like split-half validation—estimates out-of-sample, and not in-sample, effect sizes^13^. The in-sample associations^1,10^ for the method of Spisak et al.^8^ (Fig. 1b), that is, from data in the sample used to develop the model, show the same degree of overfitting (Fig. 1a versus Fig. 1b). The plot of Spisak et al.^8^ (Fig. 1c) simply adds an additional out-of-sample test (cross-validation before split half), and therefore demonstrates the close correspondence between two different methods for out-of-sample effect estimation^14^. Analogously, we can replace the cross-validation step in the code of Spisak et al.^8^ with split-half validation (our original out-of-sample test), obtaining split-half effects in the first half of the sample, and then comparing them to the split-half estimates from the full sample (Fig. 1d). The strong correspondences between cross-validation followed by split-half (Spisak et al. method^8^; Fig. 1c) and repeated split-half validation (Fig. 1d) are guaranteed by plotting out-of-sample estimates (from the same dataset) against one another. Here, plotting cross-validated discovery sample estimates on the *y* axis (Fig. 1c,d) provides no additional information beyond the *x* axis out-of-sample values. The critically important out-of-sample predictions, required for reporting multivariate results^1^, generated using the method of Spisak et al.^8^ and our method are nearly identical (Fig. 1e).

**Fig. 1:**
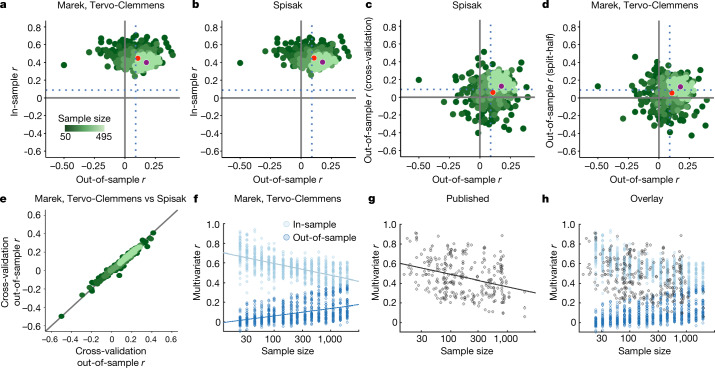
In-sample versus out-of-sample effect estimates in multivariate BWAS. **a**–**e**, Methods comparison between our previous study^1^ (split-half) and Spisak et al.^8^ (cross-validation followed by split-half). ‘Marek, Tervo-Clemmens’ and ‘Spisak’ refer to the methodolgies described in ref. ^1^ and ref. ^8^, respectively. For **a**–**e**, HCP 1200 Release (full correlation) data were used to predict age-adjusted total cognitive ability. Analysis code and visualizations (*x*,*y* scaling; colours) are the same as in Spisak et al.^8^. The *x* axes in **a**–**e** always display the split-half out-of-sample effect estimates from the second (replication) half of the data (correlation between true scores and predicted scores; as in Spisak et al.^8^ and in our previous study^1^; Supplementary Methods). **a**, In-sample (training correlation; *y* axis) as a function of out-of-sample associations (plot convention in our previous study^1^). **b**, Matched comparison of the true in-sample association (training correlations, mean across folds; *y* axis) in the method proposed by Spisak et al.^8^. **c**, The proposed correction by Spisak et al.^8^ that inserts an additional cross-validation step to evaluate the first half of data, which by definition makes this an out-of-sample association (*y* axis). **d**, Replacing the cross-validation step from Spisak et al.^8^ with a split-half validation provides a different (compared with **c**) out-of-sample association of the first half of the total data (that is, each of the first stage split halves is one-quarter of the total data; *y* axis). The appropriate and direct comparison of in-sample associations between Spisak et al.^8^ and our previous study^1^ is comparing **b** to **a**, rather than **c** to **a**. The Spisak et al. method^8^ (cross-validation followed by split-half validation) does not reduce in-sample overfitting (**b**) but, instead, adds an additional out-of-sample evaluation (**c**), which is nearly identical to split-half validation twice in a row (**d**), and makes it clear why the out-of-sample performance of these two methods is likewise nearly identical. **e**, Correspondence between out-of-sample associations (to the left-out half) from the additional cross-validation step proposed by Spisak et al.^8^ (mean across folds; *y* axis) and the original split-half validation from our previous study^1^ (*x* axis). The identity line is shown in black. **f**, In-sample (*r*; light blue) and out-of-sample (*r*; dark blue) associations as a function of sample size. Data are from figure 4a–d of ref. ^1^. **g**, Published literature review of multivariate *r* (*y* axis) as a function of sample size (data from ref. ^15^) displayed with permission. For **f** and **g**, best fit lines are displayed in log_10_ space. **h**, Overlap of **f** and **g**.

As Spisak et al.^8^ highlight, cross-validation of some type is considered to be standard practice^10^, and yet the distribution of out-of-sample associations (Fig. 1f (dark blue)) does not match published multivariate BWAS results (Fig. 1g), which have largely ranged from *r* = 0.25 to 0.9, decreasing with increasing sample size^10,15,16^. Instead, published effects more closely follow the distribution of in-sample associations (Fig. 1h). This observation suggests that, in addition to small samples, structural problems in academic research (for example, non-representative samples, publication bias, misuse of cross-validation and unintended overfitting) have contributed to the publication of inflated effects^12,17,18^. A recent biomarker challenge^5^ showed that cross-validation results continued to improve with the amount of time researchers spent with the data, and the models with the best cross-validation results performed worse on never-seen held-back data. Thus, cross-validation alone has proven to be insufficient and must be combined with the increased generalizability of large, diverse datasets and independent out-of-sample evaluation in new, never before seen data^5,10^.

The use of additional cross-validation in the discovery sample by Spisak et al.^8^ does not affect out-of-sample prediction accuracies (Fig. 1e). However, by using partial correlations and ridge regression on HCP data, they were able to generate higher out-of-sample prediction accuracies than our original results in ABCD (Fig. 2a). The five variables they selected are strongly correlated^19^ cognitive measures from the NIH Toolbox (mean *r* = 0.37; compare with the correlation strength for height versus weight *r* = 0.44)^20^ and age (not a complex behavioural phenotype), unrepresentative of BWAS as a whole (Fig. 2b (colour versus grey lines)). As the HCP is the relatively smallest and most homogeneous dataset, we applied the exact method and code of Spisak et al.^8^ to the ABCD data (Fig. 2c and Supplementary Table 2). At *n* = 1,000 (training; *n* = 2,000 total), only 31% of BWAS (44% RSFC, 19% cortical thickness) were replicable (Fig. 2d; defined as in Spisak et al.^8^; Supplementary Information). Expanding BWAS scope beyond broad cognitive abilities towards complex mental health outcomes therefore requires *n* > 1,000 (Fig. 2b–d). The absolute largest BWAS (cognitive ability: RSFC, green) reached replicability only using *n* = 400 (*n* = 200 train; *n* = 200 test) with an approximate 40% decrease in out-of-sample prediction accuracies from HCP to ABCD (Fig. 2e (lighter green, left versus right)). The methods of Spisak et al.^8^ and our previous study^1^ returned equivalent out-of-sample reproducibility for this BWAS (cognitive ability: RSFC) in the larger, more diverse ABCD data (Fig. 2e (right, dark versus light green)). Thus, the smaller sample sizes (Fig. 2b,c) that are required for out-of-sample reproducibility (Fig. 2e) reported by Spisak et al.^8^ in the HCP data did not generalize to the larger ABCD dataset. See also our previous study^1^ for a broader discussion of convergent evidence across HCP and ABCD datasets.

**Fig. 2:**
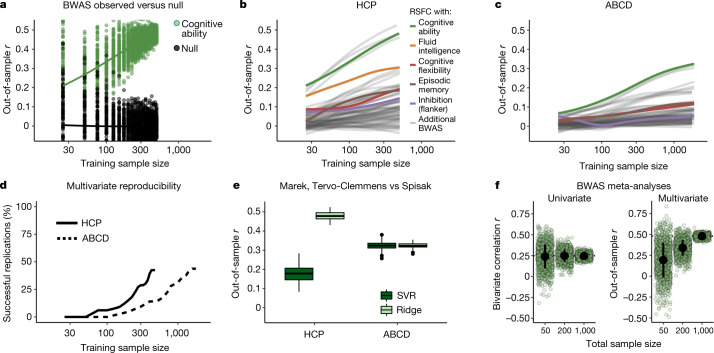
BWAS reproducibility, scope and prediction accuracy using the method of Spisak et al. **a**, Example bootstrapped BWAS of total cognitive ability (green) and null distribution (black) (*y* axis), as a function of sample size (*x* axis) from the suggested method of Spisak et al.^8^ (RSFC by partial correlation; prediction by ridge regression) in the HCP dataset (*n* = 1,200, 1 site, 1 scanner, 60 min RSFC/participant, 76% white). Sample sizes were log_10_-transformed for visualization. **b**, Out-of-sample correlation (between true scores and predicted scores) from ridge regression (*y* axis; code from Spisak et al.^8^) as a function of training sample size (*x* axis, log_10_ scaling) for 33 cognitive and mental health phenotypes (Supplementary Information) in the HCP dataset. Each line displays a smoothed fit estimate (through penalized splines in general additive models) for a brain (RSFC (partial correlations, as proposed by Spisak et al.^8^), cortical thickness) phenotype pair (66 total) that has 100 bootstrapped iterations from sample sizes of 25 to 500 (inclusive) in increments of 25 (20 total bins). Sample sizes were log_10_-transformed (for visualization) before general additive model fitting. **c**, The same as in **b**, but in the ABCD dataset (*n* = 11,874, 21 sites, 3 scanner manufacturers, 20 min RSFC/participant, 56% white) using 32 cognitive and mental health phenotypes at sample sizes of 25, 50, 75 and from 100 to 1,900 (inclusive) in increments of 100 (22 total bins). **d**, The percentage of brain–phenotype pairs (BWAS) from **b** and **c** with significant replication on the basis of the method of Spisak et al.^8^ (Supplementary Information). **e**, Comparison of our original method in our previous study^1^ and the method proposed by Spisak et al.^8^ at the full split-half sample size of HCP (left) and ABCD (right). Out-of-sample correlations (RSFC with total cognitive ability, *y* axis) for the method used in our previous study^1^ (dark green; RSFC by correlation, PCA, SVR) and by Spisak et al.^8^ (light green; RSFC by partial correlation, ridge regression). Repeating the method proposed by Spisak et al.^8^ in ABCD (right) and comparing this to the method used in our previous study^1^ results in a very similar out-of-sample *r*. **f**, Simulated individual studies (light green circles; *n* = 1,000 per sample size) and meta-analytic estimates (black dot, ±1 s.d.) using the method of Spisak et al.^8^ (partial correlations in the HCP dataset) for the largest univariate association (left; *y* axis, bivariate correlation) and multivariate association (right; *y* axis, out-of-sample correlation) for total cognitive ability versus RSFC, as a function of total sample size (*x* axis; bivariate correlation for sample sizes of 50, 200 and 1,000, and multivariate sum of train and test samples, each 25, 100 and 500). For univariate approaches, studies of any sample size, when appropriately aggregated to a large total sample size, can correctly estimate the true effect size. However, for multivariate approaches, even when aggregating across 1,000 independent studies, studies with a small sample size produce prediction accuracies that are downwardly biased relative to large sample studies, highlighting the need for large samples in multivariate analyses.

Notably, the objections of Spisak et al.^8^ raise additional reasons to stop the use of smaller samples in BWAS that were not highlighted in our original article. Multivariate BWAS prediction accuracies—absent overfitting—are systematically suppressed in smaller samples^5,9,21^, as prediction accuracy scales with increasing sample size^1,9^. Thus, the claim that “cross-validated discovery effect-size estimates are unbiased” does not account for out-of-dataset generalizability and downward bias. In principle, if unintended overfitting and publication bias could be fully eliminated, meta-analyses of small-sample univariate BWAS would return the correct association strengths (Fig. 2f (left)). However, meta-analyses of small multivariate BWAS would always be downwardly biased (Fig. 2f (right)). If we are interested in maximizing prediction accuracy, essential for clinical implementation of BWAS^22^, large samples and advancements in imaging and phenotypic measurements^1^ are necessary.

Repeatedly subsampling the same dataset, as Spisak et al.^8^ and we have done, overestimates reproducibility compared with testing on a truly new, diverse dataset. Just as in genomics^23^, BWAS generalization failures have been highlighted^5,24^. For example, BWAS models trained on white Americans transferred poorly to African Americans and vice versa (within dataset)^24^. Historically, BWAS samples have lacked diversity, neglecting marginalized and under-represented minorities^25^. Large studies with more diverse samples and data aggregation efforts can improve BWAS generalizability and reduce scientific biases contributing to massive health inequities^26,27^.

Spisak et al.^8^ worry that “[r]equiring sample sizes that are larger than necessary for the discovery of new effects could stifle innovation”. We appreciate the concern that rarer populations may never be investigated with BWAS. Yet, there are many non-BWAS brain–behaviour study designs (fMRI ≠ BWAS) focused on within-patient effects, repeated-sampling and signal-to-noise-ratio improvements that have proven fruitful down to *n* = 1 (ref. ^28^). By contrast, the strength of multivariate BWAS lies in leveraging large cross-sectional samples to investigate population-level questions. Sample size requirements should be based on expected effect sizes and real-world impact, and not resource availability. Through large-scale collaboration and clear standards on data sharing, GWAS has reached sample sizes in the millions^29–31^, pushing genomics towards new horizons. Similarly, BWAS analyses of the future will not be limited to statistical replication of the same few strongest effects in small homogeneous populations, but also have broad scope, maximum prediction accuracy and excellent generalizability.

## Reporting summary

Further information on experimental design is available in the Nature Portfolio Reporting Summary linked to this Article.

## Online content

Any methods, additional references, Nature Portfolio reporting summaries, source data, extended data, supplementary information, acknowledgements, peer review information; details of author contributions and competing interests; and statements of data and code availability are available at 10.1038/s41586-023-05746-w.

## Supplementary information

Supplementary Information Supplementary Methods, Supplementary Tables 1 and 2 and Supplementary References.

Reporting Summary

## Data Availability

Participant-level data from all datasets (ABCD and HCP) are openly available pursuant to individual, consortium-level data access rules. The ABCD data repository grows and changes over time (https://nda.nih.gov/abcd). The ABCD data used in this report came from ABCD collection 3165 and the Annual Release 2.0 (10.15154/1503209). Data were provided, in part, by the HCP, WU-Minn Consortium (principal investigators: D. Van Essen and K. Ugurbil; 1U54MH091657) funded by the 16 NIH Institutes and Centers that support the NIH Blueprint for Neuroscience Research; and by the McDonnell Center for Systems Neuroscience at Washington University. Some data used in the present study are available for download from the HCP (www.humanconnectome.org). Users must agree to data use terms for the HCP before being allowed access to the data and ConnectomeDB, details are provided online (https://www.humanconnectome.org/study/hcp-young-adult/data-use-terms).

## Code availability

Manuscript analysis code specific to this study is available at GitHub (https://gitlab.com/DosenbachGreene/bwas_response). Code for processing ABCD data is provided at GitHub (https://github.com/DCAN-Labs/abcd-hcp-pipeline). MRI data analysis code is provided at GitHub (https://github.com/ABCD-STUDY/nda-abcd-collection-3165). FIRMM software is available online (https://firmm.readthedocs.io/en/latest/release_notes/). The ABCD Study used v.3.0.14.
