# Supplementary material for: Reply to: Multivariate BWAS can be replicable with moderate sample sizes
Source: Nature. 2023 Mar 8;615(7951):E8–E12. doi: 10.1038/s41586-023-05746-w (PMC9995264; doi:10.1038/s41586-023-05746-w)
Supplement: Supplementary file 1 — Supplementary Methods, Supplementary Tables 1 and 2 and Supplementary References. [file 41586_2023_5746_MOESM1_ESM.pdf]

nature portfolio

https://doi.org/10.1038/s41586-023-05746-w

Supplementary information

Reply to: Multivariate BWAS can be replicable with moderate sample sizes

In the format provided by the authors and unedited

Supplement: Solving the BWAS reproducibility crisis requires large samples

Brenden Tervo-Clemmens[1,22] ✉, Scott Marek[2,3,22] ✉, Roselyne J. Chauvin[4], Andrew N. Van[4,5], Benjamin P. Kay[4], Timothy O. Laumann[3], Wesley K. Thompson[6], Thomas E. Nichols[7], B. T. Thomas Yeo[8,9,10,11,12], Deanna M. Barch[3,14], Beatriz Luna[15,16], Damien A. Fair[17,18,19,23] ✉ & Nico U. F. Dosenbach[2,4,5,20,21,23] ✉

[1]Department of Psychiatry, Massachusetts General Hospital, Harvard Medical School, Boston, MA, USA.
[2]Department of Radiology, Washington University School of Medicine, St Louis, MO, USA.
[3]Department of Psychiatry, Washington University School of Medicine, St Louis, MO, USA.
[4]Department of Neurology, Washington University School of Medicine, St Louis, MO, USA.
[5]Department of Biomedical Engineering, Washington University in St Louis, St Louis, MO, USA
[6]Division of Biostatistics, University of California San Diego, La Jolla, CA, USA.
[7]Oxford Big Data Institute, Li Ka Shing Centre for Health Information and Discovery, Nuffield Department of Population Health, University of Oxford, Oxford,
[8]Department of Electrical and Computer Engineering, National University of Singapore, Singapore, Singapore.
[9]Centre for Sleep and Cognition, National University of Singapore, Singapore, Singapore.
[10]Centre for Translational MR Research, National University of Singapore, Singapore, Singapore.
16 N.1 Institute for Health, Institute for Digital Medicine, National University of Singapore, Singapore, Singapore.
[11]Integrative Sciences and Engineering Programme, National University of Singapore, Singapore, Singapore.
[13]Martinos Center for Biomedical Imaging, Massachusetts General Hospital, Charlestown, MA, USA.
[14]Department of Psychological and Brain Sciences, Washington University in St Louis, St Louis, MO, USA
[15]Department of Psychology, University of Pittsburgh, Pittsburgh, PA, USA.
[16]Department of Psychiatry, University of Pittsburgh, Pittsburgh, PA, USA.
[17]Masonic Institute for the Developing Brain, University of Minnesota Medical School, Minneapolis, MN, USA.
[18]Department of Pediatrics, University of Minnesota Medical School, Minneapolis, MN, USA.
[19]Institute of Child Development, University of Minnesota Medical School, Minneapolis, MN, USA.
[20]Program in Occupational Therapy, Washington University School of Medicine, St Louis, MO, USA.
[21]Department of Pediatrics, Washington University School of Medicine, St Louis, MO, USA.

[22]These authors contributed equally: Brenden Tervo-Clemmens, Scott Marek.
[23]These authors jointly supervised this work: Damien A. Fair, Nico U. F. Dosenbach.

✉e-mail: btervo-clemmens@mgh.harvard.edu; smarek@wustl.edu; faird@umn.edu; ndosenbach@wustl.edu

Methods

Datasets

Behavioural phenotype and neuroimaging (resting-state functional connectivity, cortical thickness) data were from the Human Connectome Project HCP (HCP; 1200 Subjects Data Release) and ABCD (3.0 release; Fast Track Release 3165 collection) datasets (*n* = 3,928; see [1] for details on participant information). All HCP participants provided informed consent. The ABCD Study obtained centralised institutional review board (IRB) approval from the University of California, San Diego. Each of the 21 sites also obtained local IRB approval. Ethical regulations were followed during data collection and analysis. Parents or caregivers provided written informed consent, and children gave written assent.

Behavioural data

HCP: We used overlapping cognitive phenotypes from Spisak et al. [ref: Spisak], including age-adjusted NIH Toolbox total cognitive ability, fluid intelligence, episodic memory, cognitive flexibility, and inhibition. In addition to these five phenotypes, we explored an additional 28 behavioural phenotypes, spanning cognitive and mental health/social-emotional measures (33 phenotypes total; see Supplemental Table 1).

ABCD: We again used the same five NIH Toolbox phenotypes as in Spisak et al. (total cognitive ability, fluid intelligence, episodic memory, cognitive flexibility, and inhibition) and additional cognitive, mental health/social-emotional measures, totaling 32 behavioural phenotypes from our original paper (see Supplemental Table 2).

Across both the HCP and ABCD datasets, behavioural phenotypes were used in multivariate models of resting-state functional connectivity (RSFC) and cortical thickness (see below).

Imaging data
Two functional RSFC parcellations were used for different sets of analysis. First, following the code and analyses from Spisak et al. (https://gitlab.com/DosenbachGreene/bwas_response) the HCP-released group-ICA 100 parcels were used for analyses replicating and expanding Spisak et al.'s results in the HCP dataset, both using partial correlations (per Spisak et al.'s method [ref: Spisak]) and full, bivariate correlation (per our original work, Marek, Tervo-Clemmens et al. [1]). Second, for ABCD, replicating the original methods and analyses of our initial work (Marek, Tervo-Clemmens et al. [1]), we also used a full brain parcellation with a total of 394 regions of interest [2,3]. As above, full (bivariate) correlation and partial correlation (via inverse of Ledoit-Wolf derived covariance [4]) were used. Cortical thickness data were likewise used from the HCP S1200 release and as in our original manuscript, vertex-wise data from the ABCD Fast Track Release (3165 collection)

Behavioural Phenotype Prediction
Two supervised regression models were used: a Ridge Regression model ($\alpha = 1.0$), as proposed by Spisak et al. and a combined Principal Component Analysis (PCA) and Support Vector Regression (SVR) model, whereby half of the principal components (retaining 50% of the variance) generated from the PCA were passed as features into the SVR, as in the original work by Marek, Tervo-Clemmens et al. Both models were implemented using scikit-learn [5] in Python 3.

For both HCP and ABCD datasets, both methods (ridge regression; PCA+SVR) and using three different neuroimaging feature sets (RSFC: full correlation, partial correlation; cortical thickness), the same analyses were conducted using code directly from Spisak et al. (https://gitlab.com/DosenbachGreene/bwas_response). For each behavioural phenotype and neuroimaging feature set combination, in each dataset, a complete cases sub-dataset was compiled, removing participants with missing behavioural phenotypes or neuroimaging data. For each of these complete cases (per Spisak et al.) neuroimaging feature set behavioural phenotype sub-datasets, 100 bootstraps were run for each model. Within each bootstrap, the sub-dataset was equally and randomly split into a discovery and replication set based on a given sample size. Here, sample size is defined as the size of a sole discovery/training set (identical in size to the replication set), such that given a sample size n, the total number of participants/samples of the combined discovery and replication sets is 2*n*.

Following Spisak et al. (method and code), the discovery set was divided again into 10 cross-validation folds. However, unlike the nested cross-validation which was explored in our original manuscript and shown to not substantively change results (Marek, Tervo-Clemmens et al. [1]: Supplemental Fig. S11, S12), this procedure utilised by Spisak et al., and repeated here, did not use the additional cross-validation step for hyperparameter tuning. Rather an additional out-of-sample test was applied to the discovery dataset.

The analyses and Figures (Fig. 1, 2) in this work use combinations of Spisak et al.'s methodological suggestions and those from our original work to replicate, expand, and clarify Spisak et al.'s Matters Arising commentary and to provide a more comprehensive perspective on out-of-sample multivariate BWAS effects. Rationale and additional details for specific analyses are provided in the relevant "Main Text" and "Figure Captions". In all cases, out-of-sample associations were evaluated as the correlation between the predicted phenotype score and the true score in the out-of-sample data. In-sample (training) associations were evaluated as the correlation between the true score and the predicted score from the model developed in the discovery set (that is, the data *in the sample* used to develop the model (Fig. 1).

Successful out-of-sample replication was defined as in Spisak et al.: 80% of bootstrapped iterations for a given behavioural phenotype-brain feature set ("BWAS") that were significant (via permutation test) in the first cross-validation test are significant in the second, split half test. We note this definition of replication by Spisak et al. thus does not consider all bootstrap iterations (n = 100) run when determining replication success/failure. That is, the denominator of a replication percentage is set by the number of bootstrap iterations that are significant in the first cross-validation test. Therefore, to ensure this measure of 80% replication represented a true percentage, replication here also required that more than one bootstrap iteration (out of the total 100) replicated (as defined above). Without this criteria, the impact of sampling variability and the performance of a

single bootstrap iteration ensured that a small number of BWAS would appear to intermittently have replication successes followed by replication failure for the very smallest sample sizes. Reproducibility estimates following Spisak et al. guidelines were highly consistent with those from our original work.

Data availability

Participant level data from HCP and ABCD datasets are openly available pursuant to individual, consortium-level data access rules. The ABCD data repository grows and changes over time (https://nda.nih.gov/abcd). The ABCD data used in this report came from ABCD collection 3165 and the Annual Release 3.0, DOI 10.15154/1503209.

Data were provided, in part, by the Human Connectome Project, WU-Minn Consortium (Principal Investigators: David Van Essen and Kamil Ugurbil; 1U54MH091657) funded by the 16 NIH Institutes and Centers that support the NIH Blueprint for Neuroscience Research; and by the McDonnell Center for Systems Neuroscience at Washington University. Some data used in the present study are available for download from the Human Connectome Project (www.humanconnectome.org). Users must agree to data use terms for the HCP before being allowed access to the data and ConnectomeDB, details are provided at https://www.humanconnectome.org/study/hcp-young-adult/data-use-terms.

Code availability

Manuscript analysis code specific to this study can be found here: https://gitlab.com/DosenbachGreene/bwas_response

Code for processing ABCD and UKB data can be found here: https://github.com/DCAN-Labs/abcd-hcp-pipeline

MRI data analysis code can be found here: https://github.com/ABCD-STUDY/nda-abcd-collection-3165

Supplementary Tables

Supplementary Table 1. Human Connectome Project (HCP) psychological measures. Original HCP variable names with the corresponding descriptive labels used in the manuscript. More details on the measures can be found in the HCP wiki.

Domain	Description	HCP field
Cognition	Vocabulary	PicVocab_AgeAdj
Cognition	Attention	Flanker_AgeAdj
Cognition	Working memory	ListSort_AgeAdj
Cognition	Executive function	CardSort_AgeAdj
Cognition	Processing speed	ProcSpeed_AgeAdj
Cognition	Episodic memory	PicSeq_AgeAdj
Cognition	Reading	ReadEng_AgeAdj
Cognition	Fluid intelligence	CogFluidComp_AgeAdj
Cognition	Crystallized intelligence	CogCrystalComp_AgeAdj
Cognition	Cognitive ability	CogTotalComp_AgeAdj

Cognition	Fluid intelligence	PMAT24_A_CR
Social-emotional	Sadness	Sadness_Unadj
Social-emotional	Fear - Affect	FearAffect_Unadj
Social-emotional	Fear - Somatic Arousal	FearSomat_Unadj
Social-emotional	Anger - Affect	AngAffect_Unadj
Social-emotional	Anger - Physical Aggression	AngAggr_Unadj
Social-emotional	Anger - Hostility	AngHostil_Unadj
Social-emotional	Life Satisfaction	LifeSatif_Unadj
Social-emotional	Meaning and Purpose	MeanPurp_Unadj
Social-emotional	Positive Affect	PosAffect_Unadj
Social-emotional	Emotional Support	EmotSupp_Unadj
Social-emotional	Friendship	Friendship_Unadj
Social-emotional	Perceived Hostility	PercHostil_Unadj
Social-emotional	Perceived Rejection	PercReject_Unadj
Social-emotional	Perceived Stress	PercStress_Unadj
Social-emotional	Self Efficacy	SelfEff_Unadj
Social-emotional	Instrumental Support	InstruSupp_Unadj
Social-emotional	Loneliness	Loneliness_Unadj
Personality	Openness	NEOFAC_O
Personality	Conscientiousness	NEOFAC_C
Personality	Extroversion	NEOFAC_E
Personality	Agreeableness	NEOFAC_A
Personality	Neuroticism	NEOFAC_N

Supplementary Table 2. ABCD behavioral phenotype measures.
Original ABCD variable names with the corresponding descriptive labels used in the manuscript. More details on the demographic and psychological measures can be found in the ABCD data dictionary.

Domain	Description	ABCD field
Cognition	Vocabulary	nihtbx_picvocab_agecorrected
Cognition	Attention	nihtbx_flanker_agecorrected
Cognition	Working memory	nihtbx_list_agecorrected
Cognition	Executive function	nihtbx_cardsort_agecorrected
Cognition	Processing speed	nihtbx_pattern_agecorrected
Cognition	Episodic memory	nihtbx_picture_agecorrected
Cognition	Reading	nihtbx_reading_agecorrected
Cognition	Fluid intelligence	nihtbx_fluidcomp_agecorrected
Cognition	Crystallized intelligence	nihtbx_cryst_agecorrected
Cognition	Cognitive ability	nihtbx_totalcomp_agecorrected
Psychopathology	Anxious depressed	cbcl_scr_syn_anxdep_t
Psychopathology	Withdrawn depressed	cbcl_scr_syn_withdep_t
Psychopathology	Somatic complaints	cbcl_scr_syn_somatic_t
Psychopathology	Social problems	cbcl_scr_syn_social_t
Psychopathology	Thought problems	cbcl_scr_syn_thought_t
Psychopathology	Attention problems	cbcl_scr_syn_attention_t
Psychopathology	Rule-breaking behavior	cbcl_scr_syn_rulebreak_t
Psychopathology	Aggressive behavior	cbcl_scr_syn_aggressive_t
Psychopathology	Internalizing	cbcl_scr_syn_internal_t
Psychopathology	Externalizing	cbcl_scr_syn_external_t
Psychopathology	Psychopathology	cbcl_scr_syn_totalprob_t
Psychopathology	Psychosis symptoms	pps_y_ss_number
Psychopathology	Psychosis severity	pps_y_ss_severity_score
Personality	Behavioral inhibition	bis_y_ss_bis_sum
Personality	Reward responsiveness	bis_y_ss_bas_rr
Personality	Drive	bis_y_ss_bas_drive

Personality	Fun seeking	bis_y_ss_bas_fs
Personality	Negative urgency	upps_y_ss_negative_urgency
Personality	Positive urgency	upps_y_ss_positive_urgency
Personality	Lack of planning	upps_y_ss_lack_of_planning
Personality	Lack of perseverance	upps_y_lack_of_perseverance
Personality	Sensation seeking	upps_y_ss_sensation_seeking

References

1. Marek, S. *et al.* Reproducible brain-wide association studies require thousands of individuals. *Nature* (2022) doi:10.1038/s41586-022-04492-9.
2. Gordon, E. M., Laumann, T. O. & Adeyemo, B. Generation and evaluation of a cortical area parcellation from resting-state correlations. *Cerebral* (2016).
3. Seitzman, B. A. *et al.* A set of functionally-defined brain regions with improved representation of the subcortex and cerebellum. *Neuroimage* **206**, 116290 (2020).
4. Ledoit, O. & Wolf, M. A well-conditioned estimator for large-dimensional covariance matrices. *J. Multivar. Anal.* **88**, 365–411 (2004).
5. Pedregosa, Varoquaux & Gramfort. Scikit-learn: Machine learning in Python. *of machine Learning …* (2011).

Author contributions

Conception: B.T.-C., S.M., D.A.F. and N.U.F.D. Design: B.T.-C., S.M., R.J.C., D.A.F. and N.U.F.D. Data acquisition, analysis and interpretation: B.T.-C., S.M., R.J.C., A.V.N., B.P.K., W.K.T., T.E.N., B.T.T.Y., D.A.F. and N.U.F.D. Manuscript writing, revising: B.T.-C., S.M., R.J.C., A.V.N., B.P.K., T.O.L., W.K.T., T.E.N., B.T.T.Y., D.M.B., B.L., D.A.F. and N.U.F.D
